# Prevalence of obsessive-compulsive disorder (OCD) among Iraqi undergraduate medical students in time of COVID-19 pandemic

**DOI:** 10.1186/s43045-021-00086-9

**Published:** 2021-02-08

**Authors:** Taqi Mohammed Jwad Taher, Shaymaa Abdul Lateef Al-fadhul, Ali A. Abutiheen, Hasanain Faisal Ghazi, Naeem Shami Abood

**Affiliations:** 1 Department of Family and Community Medicine, College of Medicine, Wasit University, Kut, Iraq; 2 Department of Family and Community Medicine, College of Medicine, University of Kufa, Kufa, Iraq; 3 Department of Family and Community Medicine, College of Medicine, University of Kerbala, Kerbala, Iraq; 4 College of Nursing, Al-Bayan University, Baghdad, Iraq; 5 Department of Psychiatry, Al-Zahraa Teaching Hospital, Wasit Health Directorate, Kut, Iraq

**Keywords:** Obsessive-compulsive disorder (OCD), COVID-19 pandemic, Medical students, OCD symptoms, Iraq

## Abstract

**Background:**

Obsessive-compulsive disorder (OCD) is a common mental disorder affecting millions of people. Its onset and peak are during late teens making university students a priority target. Medical student perhaps is at greater risk for developing OCD while corona COVID-19 is expected to more exaggerate symptoms mainly with extra hygiene and cleanliness practices. The aim of the study was to estimate the prevalence of OCD symptoms among Iraqi medical students, and to assess the associated psychological symptoms and its correlates. An analytic cross-sectional study was conducted among Iraqi medical students during the period from August to October 2020. An online anonymous, voluntary, and self-administered questionnaire based on the 18 questions Obsessive-Compulsive Inventory-Revised scale (OCI-R) was used to collect the data.

**Results:**

A total of 1644 students had filled the questionnaire. Females were 1116 (67.9%), while 1153 (70.1%) had reported accompanying mental symptoms. Of which worry and stress were the most prevalent with 674 (25.9%) and 617 (23.7%) respectively. However, 707 (43%) have probable OCD symptoms that need further assessment. Unpleasant thoughts were the most prevalent symptoms with 51.8%. Surprisingly, the washing and contamination scales were low at 14% and 19.4% while repeating certain numbers was the least with 8%. OCD symptoms were significantly related to younger age and earlier years of study. Further, all accompanied mental symptoms were significantly associated with probable OCD status.

**Conclusion:**

High prevalence of OCD among medical students during the COVID-19 pandemic. No association of OCD with gender and family history. Younger students and early years of study were more likely to suffer from OCD symptoms.

## Background

Obsessive-compulsive disorder (OCD) is a chronic psychiatric disorder. It causes unwanted thoughts (obsessions) and repetitive actions (compulsions). In their everyday lives, many people have concentrated on ideas and repetitive acts. But, for people with OCD, the thoughts are more recurring, and the repetitive behaviors are over and cannot be ignored that they can disrupt their life [1–4].

Obsessive-compulsive disorder (OCD) is reported to be the fourth most frequent mental disorder worldwide [5, 6]. OCD prevalence varies over age, regions, and others. However, OCD lifetime prevalence is estimated to be 2.3% ranging from 1.1-3.3%. Some researchers indicated that OCD affects females slightly higher than males but other does not [1, 2, 3, 7, 8].

Patients with OCD can have one or more groups of OCD symptoms. These symptoms can include an obsession with contamination washing rituals, frequent checking, persistent nasty or religious thoughts, redundant hoarding, uniformity and ordering, and others. However, these symptoms and attitudes are unsteady and tend to disappear for a period and return or change in their categories throughout the disease [7, 9, 10, 11].

While the exact causes of OCD are unclear and vary between genetic, environmental, and neurobiological factors. Patients with OCD might be with other co-existing mental hazards such as anxiety disorder, post-traumatic stress disorder, depression, substance use, eating disorder, learning, and suicidal thoughts [2, 8, 12].

Diagnosis of OCD is so difficult, not just because of the overlap of symptoms with other psychiatric disorders and diseases. But also, because many patients deny and not confirm the symptoms and ill behaviors they have encountered for numerous reasons, including the stigmatization of such mental diseases. And this raises the need for screening for OCD and OCD symptoms among patients with other mental disorders and risky groups including university students [2, 13, 14].

As the onset of OCD develops mainly during adolescence and late teens with a median age of onset of 19-20 years. That makes high school and university students a remarkable target for screening for OCD and OCD symptoms. Further, studies reported double rates of OCD among university students in comparison to their rates among the general population. Furthermore, this age group is more prone to other mental hazards including substance use and suicidal attempts that are explained to be associated comorbidities for OCD [2, 13, 15, 16].

Undergraduate Medical students are at an increased risk for OCD, due to the stressful nature of medical schools as part of the heavy curriculum, less leisure time, as well that medical students are taught, trained, and asked to be more precise, perfect, and obsessive a little bit more. On the other hand, OCD can adversely affect academic performance, general well-being, social interaction, and suicidal thoughts. Issues that might have a huge impact on life unless being diagnosed early and properly managed [4, 6, 11, 12, 17].

Corona COVID-19 pandemic is the biggest health challenge faced by the world in recent history. Affecting most countries with more than 42 million registered cases and over 1.150.000 deaths reported death till October 25. Iraq reported the highest number of cases and deaths among Arab countries, with more than 450 thousand cases and more than 10 thousand deaths.

Corona COVID-19 pandemic is expected to have a negative effect on OCD patients and medical students firstly as a general stressor on health and communities, secondly by the closure of medical schools and the shifting toward online learning, and lastly by the increasing efforts of handwashing and general hygiene as an essential step in COVID-19 prevention which might trigger the obsession with contamination and compulsive washing of hands. Which are reported as the most common symptoms of OCD [18, 19, 20].

So, this study aims to estimate the prevalence of the suggestive symptom of obsessive-compulsive disorder (OCD) among a sample of medical students in Iraqi universities during the COVID-19 pandemic era and to assess the associated psychological symptoms. In addition to the association of the probability of being OCD with the related sociodemographic features and other psychological symptoms.

## Methods

An analytic cross-sectional study. This study was conducted among Iraqi medical students during the period from August 2020 to October 2020. The population of the study included all medical students attending different Iraqi universities except Kurdistan (north of Iraq). A convenient sample was included in this study. Data were collected by a structured questionnaire submitted online through different groups for medical students including Facebook, telegram, and WhatsApp groups for 2 weeks period. The questionnaire consisted of three parts: the first was containing sociodemographic features and associated risk factors, the second part consisted of 18 items from the Obsessive-Compulsive Inventory-Revised scale (OCI-R) [21], and the third part contains the summation of the final score of the participant. The questionnaire was translated from English to the Arabic language by a language specialist and pretested on 25 medical students who were excluded from the final analysis.

The OCI-R is a reduced form of the original OCI that consisted of 18 subjective questions that can be used for screening of OCD but it never gives a definite diagnosis. It can define the probable cases during the last month. The answer for each question consisted of the Likert scale (not at all, a little, moderately, a lot, and extremely) which coded from 0-4 respectively with a maximum total of 72, score more than 27 was considered as a probable OCD [4].

All 18 questions are used for the assessment of six types of symptoms related to OCD, including checking, hoarding, neutralizing, obsessing, ordering, and washing. The Arabic version for OCI-R was tested for validity and reliability in a previous study conducted in Saudi Arabia [22].

An official agreement was taken from the ethical approval committee. All participant students were consented to participate in a questionnaire filling with confidentiality. Students had the ability to scoring their symptoms to know if they are probably having OCD to be diagnosed as early as possible and seek medical advice and treatment.

Data were entered and analyzed by SPSS version 23. Descriptive statistics were used by the mean and standard deviation for numerical data, while categorical data were represented by frequency tables. Association between categorical variables was performed by the chi-square test and the difference between means was performed by *t* test for independent samples considering *p* value equal or less than 0.05 as significant.

## Results

The total sample size involved in this study was 1644 medical students who were willing to participate in this survey and answered the questionnaire through 2 weeks period from 19 August-2 September 2020. The sociodemographic features of participants and family history are presented in Table 1.

**Table 1:** Frequency distribution of socio-demographic features of the study participants

**Categorical variables**	**Frequency**	**Percent**
**Gender**		
Male	528	32.1
Female	1116	67.9
**Marital status**		
Single	1586	96.5
Married	58	3.5
**College stages**		
First stage	531	32.3
Second stage	327	19.9
Third stage	336	20.4
Fourth stage	199	12.1
Fifth stage	133	8.1
Sixth stage	118	7.2
**Place of living**		
Baghdad	186	11.3
Wasit	285	17.3
Karbala	333	20.3
Thi-qar	111	6.8
Nineveh	2	0.1
Qadisiyyah	60	3.6
Babil	82	5.0
Najaf	425	25.9
Muthanna	19	1.2
Maysan	6	0.4
Diyala	19	1.2
Basra	103	6.3
Al Anbar	6	0.4
Saladin	3	0.2
Sulaymaniyah	2	0.1
Erbil	1	0.1
Kirkuk	1	0.1
**Family history of OCD**		
No	1291	78.5
Yes	353	21.5
**Continuous variables**	**Mean**	**Standard deviation**
**Age of participants (years)**	20.73	1.83
**Number of sleep hours**	8.11	1.85

According to the results mentioned in Table 1, more than two-thirds (67.9%) of the participants were females, only 3.5% were married, around one-third (32.3%) are from the first stage. The higher response rate was among students living in Najaf and Karbala (25.9% and 20.3%) respectively. Less than one quarter (21.5%) mentioned that they have a family member suffering from OCD.

The psychological and mental symptoms were reported from 1153 (70.1%) of the participants as shown in Table 2. The majority of students mentioned symptoms of worry and stress in 58.5% and 53.5% in the same order.

**Table 2:** Frequency distribution of other psychological symptoms among the study sample

Associated psychological symptoms	Responses	
	***N***	Percent
**Worry**	674	25.9%
**Depression**	465	17.9%
**Drug abuse**	2	0.1%
**Sleeping disorders**	476	18.3%
**Feeding disorders**	283	10.9%
**Stress**	617	23.7%
**Other**	84	3.2%
**Total**	2601	100.0%

**Fig. 1:**
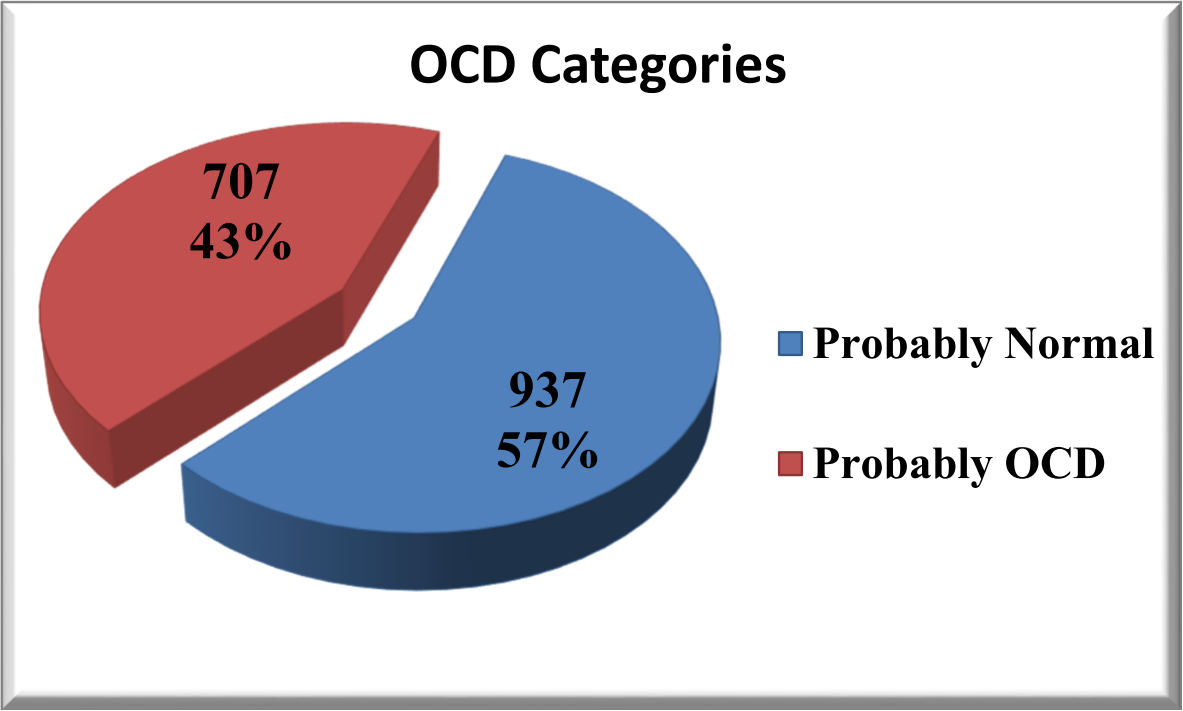
Frequency distribution of OCD categories among participant students

According to OCI-R, the mean score for all students is found as (27.3 ± 12.4). There were 937 (57%) of them probably normal persons, while 707 (43%) of them were probably having OCD and need for further assessment for a definite diagnosis as shown in Fig. 1.

The frequency distribution of student’s answers for the 18 items related to OCI-R score was presented in Table 3. Regarding the items that represented obsessing symptoms, the highest percentage of students answered, “extremely or a lot of” was 51.8% for the item “I am upset by unpleasant thoughts that come into my mind against my will” followed by 42% for the item “I frequently get nasty thoughts and have difficulty in getting rid of them.” There were 29.6% of students said that they were extremely, or a lot of times check things more often than necessary which represented the higher percentage among items related to checking. About 28% of students mentioned they suffered from hoarding symptoms related to OCD which is “I avoid throwing things away because I am afraid, I might need them later.” Near half (40.9) of students were extremely or a lot upset if objects are not arranged properly which is one of the ordering subscales of OCI-R. Only 19.4% of the students had to wash or clean themselves simply because they feel they were contaminated (washing subscale). For the neutralizing subscale of OCI-R, there were only 15.9% of students extremely or a lot of felt that there are good and bad numbers. The mean ± SD for each of the hoarding, checking, ordering, neutralizing, washing, and obsessing symptoms were 1.2 ± 0.8, 1.5 ± 0.9, 1.9 ± 0.9, 0.9 ± 0.8, 1.2 ± 0.9, and 2.0 ± 1.1 in the same order.

**Table 3:** Frequency distribution of the participant’s answers for OCI-R score items

Questions	Not at all	A little	Moderately	A lot	Extremely
	No. (%)	No. (%)	No. (%)	No. (%)	No. (%)
I have saved up so many things that they get in the way.	592 (36)	559 (34)	343 (20.9)	138 (8.4)	12 (0.7)
I check things more often than necessary.	102 (6.2)	409 (24.9)	482 (29.3)	481 (29.3)	170 (10.3)
I get upset if objects are not arranged properly.	158 (9.6)	389 (23.7)	424 (25.8)	505 (30.7)	168 (10.2)
I feel compelled to count while I am doing things.	751 (45.7)	382 (23.2)	283 (17.2)	189 (11.5)	39 (2.4)
I find it difficult to touch an object when I know it has been touched by strangers or certain people.	557 (33.9)	538 (32.7)	280 (17)	189 (11.5)	80 (4.9)
I find it difficult to control my own thoughts.	267 (16.2)	491 (29.9)	334 (20.3)	358 (21.8)	194 (11.8)
I collect things I do not need.	571 (34.7)	568 (34.5)	319 (19.4)	158 (9.6)	28 (1.7)
I repeatedly check doors, windows, drawers, etc.	512 (31.1)	486 (29.6)	344 (20.9)	214 (13)	88 (5.4)
I get upset if others change the way I have arranged things.	197 (12)	454 (27.6)	328 (20)	451 (27.4)	214 (13)
I feel I have to repeat certain numbers.	933 (56.8)	350 (21.3)	229 (13.9)	97 (5.9)	35 (2.1)
I sometimes have to wash or clean myself simply because I feel contaminated.	449 (27.3)	483 (29.4)	393 (23.9)	236 (14.4)	83 (5)
I am upset by unpleasant thoughts that come into my mind against my will.	159 (9.7)	378 (23)	255 (15.5)	502 (30.5)	350 (21.3)
I avoid throwing things away because I am afraid I might need them later.	275 (16.7)	439 (26.7)	469 (28.5)	352 (21.4)	109 (6.6)
I repeatedly check gas and water taps and light switches after turning them off.	622 (37.8)	478 (29.1)	282 (17.2)	185 (11.3)	77 (4.7)
I need things to be arranged in a particular way.	252 (15.3)	460 (28)	520 (31.6)	332 (20.2)	80 (4.9)
I feel that there are good and bad numbers.	737 (44.8)	358 (21.8)	288 (17.5)	189 (11.5)	72 (4.4)
I wash my hands more often and longer than necessary.	614 (37.3)	425 (25.9)	408 (24.8)	140 (8.5)	57 (3.5)
I frequently get nasty thoughts and have difficulty in getting rid of them.	183 (11.1)	464 (28.2)	308 (18.7)	407 (24.8)	282 (17.2)

There was a significant association between both groups of students with college class (*p* < 0.001) and significant differences with age (*p* < 0.02). The presence of other psychological symptoms like worry, depression, sleeping disorders, feeding disorders, and stress was significantly associated with the probable OCD (*p* < 0.001). More than one-third of students (34.5%) of the probable OCD group were from the first medical year. About 50% and 46.5% of students with symptoms suggesting OCD was suffering from worry and stress respectively.

## Discussion

This is one of the scarce worldwide studies conducted for assessment of OCD and investigation of associated psychological symptoms, and sociodemographic features (Table 4). To our knowledge, there are no previous studies conducted in Iraq for estimation of the prevalence of OCD among the general population, meanwhile medical students during the COVID-19 outbreak. Current results showed that more than two-thirds of the responders are females, and this agrees with a similar female predominance in medical colleges. The prevalence of probable OCD in this sample is 43%. This is higher than the prevalence reported from a previous study conducted on 200 medical students at the University of Sulaimani in Iraq, which was 6% [23]. Earlier studies conducted among medical students in different countries reported lower prevalence ranged from 3.8 to 35.8% [4, 9, 24]. The current high prevalence is in congruence with an online survey of 6041 Canadian participants, in which a significantly higher prevalence of OCD symptoms during COVID-19 pandemic by 10-13 folds than pre-pandemic period [25].

**Table 4:** Frequency distribution and association of probably normal and OCD among different socio-demographic features

Variables	Probably normal students (***N*** = 937)	Probably OCD students (***N*** = 707)	***p*** value
**Age\years (mean ± SD)**	20.8 ± 1.8	20.5 ± 1.7	**0.002**
**Numbers of sleep hours (mean ± SD)**	8.1 ± 1.7	8.1 ± 2.0	0.81
**Gender**	***N*** **(%)**	***N*** **(%)**	
Males	312 (33.3)	216 (30.6)	0.23
Females	625 (66.7)	491 (69.4)	
**Marital status**			
Single	902 (96.3)	684 (96.7)	0.6
Married	35 (3.7)	23 (3.3)	
**College years**			
First year	287 (30.6)	244 (34.5)	**< 0.001**
Second year	169 (18)	158 (22.3)	
Third year	177 (18.9)	159 (22.5)	
Fourth year	136 (14.5)	63 (8.9)	
Fifth year	88 (9.4)	45 (6.4)	
Sixth year	80 (8.5)	38 (5.4)	
**Family history of OCD**			
Yes	186 (19.9)	167 (23.6)	0.06
No	751 (80.1)	540 (76.4)	
**Presence of other psychological symptoms**			
Worry	319 (34)	355 (50.2)	**< 0.001**
Depression	212 (22.6)	253 (35.8)	**< 0.001**
Drug abuse	0 (0)	2 (0.3)	0.1
Sleeping disorders	227 (24.2)	249 (35.2)	**< 0.001**
Feeding disorders	126 (13.4)	157 (22.2)	**< 0.001**
Stress	288 (30.7)	329 (46.5)	**< 0.001**

Concerning associated psychological symptoms, lots of students reported having different psychological symptoms including worry then stress, sleeping disorders, and depression. Similar to current results, studies conducted in China, and Italy [26–28] indicated moderate to severe psychological impact of the COVID-19 pandemic with a higher rate of distress, anxiety, and depression among participants.

It was found that probable OCD was significantly associated with symptoms of either of worry, stress, depression, sleeping, and feeding disorders (*p* < 0.001). Correlation of OCD with stress, anxiety, and depression symptoms was reported by previous similar studies [4, 24, 25]. People with OCD often have other psychological comorbidities. Many of them have a lifetime diagnosis of a depressive or bipolar disorder (63%) or an anxiety disorder (76%) [29].

At this point, we should consider the role of COVID-19 as stressors that lead to a higher prevalence of OCD as well as other psychological symptoms, in addition to that medical students have a relatively higher prevalence of mental health problems than the general population as reported by meta-analytic studies [30, 31].

Current results showed that probably OCD students significantly younger than probably normal students (*p* < 0.002), meanwhile more than one-third (34.5%) of probably OCD students were from the first stages. Statistically, there is a highly significant association between OCD and college years (*p* < 0.001); this aligns with the previous study conducted among Brazilian medical students [4]; the authors demonstrated that severe obsessive-compulsive symptoms were associated with being in the first year, having depressive symptoms and difficulty in adaptation. Whereas other studies [9, 23] showed a non-significant relationship between age and OCD.

In this study, statistical analysis showed that there was no significant gender difference according to the presence of OCD, although females had higher than males with probable OCD (69.4% vs 30.6%). Current results on gender are consistent with the findings of other researchers [4, 9, 24].

Considering the presence of a family history of OCD, 23.6% of probable OCD students reported positive family history versus 19.9% of probably normal students; however, a non-significant association was found between family history and OCD frequency. This is in contrast to the finding of other authors [9, 23] who stated that a family history of OCD was more reported from students with OCD than from normal ones. This finding could be explained by the presence of additional environmental stressors which were triggered recent OCD cases rather than genetic factors. The results of this study showed a higher mean of OCD symptoms among the participants was an obsession. Likewise, Jaisoorya et al. [8] found a higher proportion of Indian college students with OCD reported obsessions. Whereas Torres et al. [4] reported a higher mean of OCD student was an ordering.

### Limitations

This study had some limitations. First, the diagnosis of OCD depended on the self-reported responses of students. No clinical interview was followed to confirm the diagnosis. Second, this study was cross-sectional in design; therefore, the causal association cannot be ascertained. Third, the sampling method was convenient; this may limit the generalization of results. Lastly, the pandemic cannot be confirmed as the exact causes of OCD, so that longitudinal researches are necessary for confirming the pandemic effect on the psychological well-being of students. However, regardless of these limitations, the current study included a large sample size from different Iraqi cities; a well-validated instrument had been used to minimize information bias. The prevalence of OCD and its associated factors was evaluated for the first time during the COVID-19 outbreak among medical students.

## Conclusion

This study demonstrates a high prevalence of OCD among medical students during the COVID-19 outbreak. Students with OCD have significant psychological symptoms. The results suggest no association of OCD with gender and family history. Younger students are more likely to suffer from OCD. Symptoms of OCD often hidden, negatively influence many life aspects of these students, therefore, efforts should be made by the college deanery to early identify and treat this condition. Further studies are recommended to measure the psychological impact of the pandemic on other college students and the general population.

## Data Availability

Available on request

## Abbreviations

OCD: Obsessive-compulsive disorder
OCI-R: Obsessive-Compulsive Inventory-Revised scale
COVID-19: Coronavirus disease
